# Aqueous Ionic Liquid Mixtures as Minimal Models of Lipid Bilayer Membranes

**DOI:** 10.1021/acsbiomaterials.4c00740

**Published:** 2024-07-27

**Authors:** Jonas Volmer, Ulrike Cerajewski, Marie Alfes, Julian Bender, Josefin Abert, Carla Schmidt, Maria Ott, Dariush Hinderberger

**Affiliations:** † Martin Luther University Halle-Wittenberg, Institute of Chemistry, Physical Chemistry − Complex Self-Organizing Systems, Von-Danckelmann-Platz 4, 06120 Halle (Saale), Germany; ‡ Interdisciplinary Research Centre HALOmem, Institute of Biochemistry and Biotechnology, Charles Tanford Protein Centre, Martin Luther University Halle-Wittenberg, Kurt-Mothes-Str. 3a, 06120 Halle, Germany; § Department of Chemistry − Biochemistry, Johannes Gutenberg University Mainz, Biocenter II, Hanns-Dieter-Hüsch-Weg 17, 55128 Mainz, Germany; ∥ Martin Luther University Halle-Wittenberg, Institute of Biochemistry and Biotechnology, Protein Biochemistry, Kurt-Mothes-Str. 3, 06120 Halle (Saale), Germany

**Keywords:** Ionic Liquids, Pseudo-Membrane Systems, Myelin Basic Protein, ESR/EPR Spectroscopy, SAXS, Mass Spectrometry, ATR-IR Spectroscopy, Dynamic Light Scattering, Protein Folding

## Abstract

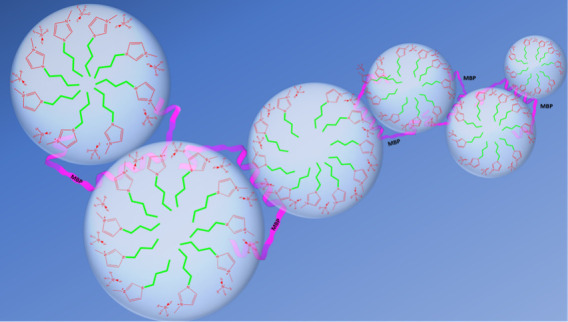

We introduce aqueous ionic liquid (IL) mixtures, specifically mixtures of 1-butyl-3-imidazoliumtetrafluoroborate (BMImBF_4_), with water as a minimal model of lipid bilayer membranes. Imidazolium-based ILs are known to form clustered nanoscale structures in which local inhomogeneities, micellar or lamellar structures, are formed to shield hydrophobic parts of the cation from the polar cosolvent (water). To investigate these nanostructures, dynamic light scattering (DLS) on samples with different mixing ratios of water and BMImBF_4_ was performed. At mixing ratios of 50% and 45% (v/v), small and homogeneous nanostructures can indeed be detected. To test whether, in particular, these stable nanostructures in aqueous mixtures may mimic the effects of phospholipid bilayer membranes, we further investigated their interaction with myelin basic protein (MBP), a peripheral, intrinsically disordered membrane protein of the myelin sheath. Using dynamic light scattering (DLS), continuous wave (CW) and pulse electron paramagnetic resonance (EPR), and small-angle X-ray scattering (SAXS) on recombinantly produced, “healthy” charge variants rmC1WT and double cysteine variant C1S17CH85C, we find that the size and the shape of the determined nanostructures in an optimum mixture offer model membranes in which the protein exhibits native behavior. SAXS measurements illuminate the size and shape of the nanostructures and indicate IL-rich “beads” clipped together by functional MBP, one of the in vivo roles of the protein in the myelin sheath. All the gathered data combined indicate that the 50% and 45% aqueous IL mixtures can be described as offering minimal models of a lipid mono- or bilayer that allow native processing and potential study of at least peripheral membrane proteins like MBP.

## Introduction

The history of ionic liquids is about 150 years old, but due to their advantageous properties such as non- or low flammability, low toxicity, negligible vapor pressure, and their ensuing lower environmental impact, they are often, maybe too often, referred to as “green solvents”. Their apparent uses have thus sparked scientific interest over the last three decades.^1−3^ They can be used to catalyze chemical reactions as an electrolyte or as a substitute for conventional organic solvents. Despite these outstanding properties and possible uses, the application of ionic liquids still vastly lags behind the fundamental research work and their potential is still largely unexplored, e.g., in pharmaceutical applications.^1−4^

In the realm of protein science, intrinsically disordered proteins (IDPs) have for the past two decades shifted and expanded the view of the structure–function paradigm. IDPs are highly specific biological macromolecules that perform essential tasks in vivo but are not crystallizable and do not form tertiary structures.^5−8^ IDPs play important roles in physiological processes such as signaling, cell communication, and chaperone activity. They can coarsely be divided into two categories, (fully) intrinsically disordered proteins (IDPs) and proteins with intrinsically disordered regions (IDRs).^9−11^ Because of their involvement in physiological and pathological processes, intrinsic flexibility as evidenced in, e.g., disorder-to-order-transitions upon binding ligands, is of great interest.^12^ IDPs can also be highly specific pharmaceutical target proteins that change their conformation at their destination or in the vicinity of their target ligand.^10,12^

This work attempts to unite these two so far relatively disjointed areas by investigating the influence of ionic liquids on structure and dynamics of intrinsically disordered proteins.^13^ During the research work in this project, it became apparent there might be new fields of application for ILs in biophysical chemistry, in particular for the in vitro treatment (purification, reconstitution, etc.) and for the study of phospholipid-membrane bound proteins. While the influence of ILs on the structure and dynamics of soluble proteins has been in the focus of the scientific community for some time, their effect on membrane-based proteins is still mainly unknown.^13^

Here, the myelin basic protein (MBP) was chosen as the model protein. MBP is an IDP of the myelin sheath of vertebrates, which ensures compaction of this multilamellar sheath and as such guarantees and expedites unhindered signal transmission between nerve cells. Posttranslational modification (deamination) of MBP leads to different isoforms that in particular vary in net charge and charge distribution, which seems to be at least correlated with the progression of demyelinating diseases such as multiple sclerosis (MS) and has therefore been in the focus of research in recent decades.^14−19^

1-Butyl-3-methylimidazolium tetrafluoroborate (BMIm) was chosen as the model IL for two reasons. In previous studies, imidazole-based ILs were found to be interesting in their effects on the native structure of (solution-based) proteins.^12,20,21^ Moreover, with a butyl group attached at the imidazolium cation, the alkyl chain can be seen as being representative of many “standard” ILs with a medium-sized butyl chain as opposed to ILs containing much more hydrophobic moieties.^3,20^ Dynamic light scattering (DLS) was chosen as a simple, fast, and reliable method to obtain insights into self-assembly on the nanometer scale.^22−24^ To obtain additional, more detailed information about the “internal” structure and dynamics of the protein or the IL nanostructures, methods such as infrared spectroscopy (IR), electron spin resonance (ESR/EPR) spectroscopy, and small-angle X-ray scattering (SAXS) were used.^25−27^

## Materials and Methods

### CW EPR Spectroscopy

First, ∼10 μL of the sample solutions were filled into 50 μL microcapillaries and then sealed with CRITOSEAL. After 12 h of incubation at 65 °C, the samples were measured at 37 °C in a Miniscope MS400 (Magnettech GmbH, Berlin, now part of Bruker BioSpin) benchtop EPR spectrometer. The frequency was set to ∼9.4 GHz, the magnetic center field B_0_ was set to 336 mT, the sweep width to 150 mT, the sweep time to 60 s, the modulation amplitude to 0.05 mT, the microwave (MW) power to 20 mW, and the phase to 180°. Ten scans were always recorded with 4096 measuring points.^28,29^

### Pulse EPR Spectroscopy

All four-pulse DEER (double electron–electron resonance) experiments were performed on an ElexSys E580 spectrometer (BRUKER BioSpin) equipped with an ER4118X-MS3 flexline split ring resonator and an ARS AF204 closed cycle cryostat (custom-made, ARS, Macungie, USA). A 100 μL portion of the sample was filled into an X-band tube and shock-frozen in supercooled 2-methylbutane. Visual inspection of the samples after freezing as well as the good EPR signal that indicates that all spin labels are properly solvated indicate homogeneous glass formation. The frozen sample was placed in the resonator, and a simple electron spin echo (ESE) experiment was performed. All pulse experiments were performed at X-band frequencies of 9.3–9.6 GHz and 50 K. For the DEER experiment, the pump frequency ν_pump_ was set to ESE and the observation frequency ν_obs_ was set at the low field maximum.^30,31^

### Dynamic Light Scattering

Dynamic light scattering measurements were performed on a Litesizer 500 apparatus (Anton Paar GmbH, Graz, Austria). In each case, 45 μL of the sample solution were pipetted into a low-volume quartz cuvette (Hellma Analytics, Muellheim, Germany). A temperature series of 20, 25, 30, 35, and 37 °C was measured for all samples at a detection angle of 90° (side scattering) and 175° (back scattering). The measurements were set with Kalliope to six runs of 30 s each with an equilibration time of 1 min between the temperatures. The meter focus and filter settings were set to automatic. A tight analysis model and a cumulant model were used for data analysis.^23^

### Protein Identification by LC-MS/MS

Protein gel bands were excised, and the proteins were hydrolyzed as described previously.^32^ In brief, proteins were reduced with 10 mM dithiothreitol, alkylated with 55 mM iodoacetamide, and hydrolyzed with trypsin (Roche).^32^ Extracted peptides were dissolved in 2% (v/v) acetonitrile and 0.1% (v/v) formic acid and separated using a DionexUltiMate 3000 RSLCnano System (Thermo Fisher Scientific). For this, the peptides were first loaded onto a reversed-phase C18 precolumn (μ-Precolumn C18 PepMap 100, C18, 300 μm I.D., 5 μm pore size; Thermo Fisher Scientific). Then, 0.1% formic acid (v/v) was used as mobile phase A and 80% (v/v) acetonitrile and 0.1% (v/v) formic acid were used as mobile phase B. The peptides were then separated on a reversed-phase C18 analytical column (HPLC column Acclaim PepMap 100, 75 μm I.D., 50 cm, 3 μm pore size; Thermo Fisher Scientific) with a gradient of 4–90% B over 70 min at a flow rate of 300 nl min^–1^. Peptides were directly eluted into a Q Exactive Plus Hybrid Quadrupole–Orbitrap mass spectrometer (Thermo Fisher Scientific). Data acquisition was performed in data-dependent and positive ion mode. Mass spectrometric conditions were as follows: capillary voltage, 2.8 kV; capillary temperature, 275 °C; normalized collision energy, 30%; MS scan range in the Orbitrap, *m*/*z* 350–1600; MS resolution, 70,000; automatic gain control (AGC) target, 3e6. The 20 most intense peaks were selected for fragmentation in the HCD cell at an AGC target of 1e5. MS/MS resolution, 17,500. Previously selected ions were dynamically excluded for 30 s and singly charged ions and ions with unrecognized charge states were also excluded. Internal calibration was performed using the lock mass *m*/*z* 445.120025.^33^

For protein identification, raw data were searched against a reduced database containing the protein sequences as well as contaminant sequences using MaxQuant v1.6.3.4 with the following database search settings: enzyme, trypsin; mass accuracy of precursor ions in main search, 4.5 ppm; MS/MS mass tolerance, 0.5 Da; number of allowed missed cleavages, 2; variable modifications, oxidation of methionine, carbamidomethylation of cysteine, and acetylation of protein N-terminus; FDR, 1%.^34,35^

### Native Mass Spectrometry

30 microliters of 2 mg/mL bMBP (Sigma-Aldrich) in phosphate buffer (10 mM phosphate buffer, 2.7 mM KCL, 137 mM NaCl, pH 7.4) were transferred into 200 mM ammonium acetate solution using Micro Bio-Spin 6 size-exclusion chromatography units (Bio-Rad). The eluate was loaded into gold-coated glass capillaries prepared in-house and subsequently injected into a Synapt G1 (Waters Corp.) quadrupole time-of-flight mass spectrometer modified for the transmission of high masses (MSVision). Analysis parameters were as follows: capillary voltage, 1.5 kV; sampling cone, 120 V; extraction cone, 5 V; backing pressure, 5–8 mbar; trap and transfer collision energy, 15 V; IMS pressure, 1.3 × 10^–3^ mbar. Mass spectra were externally calibrated using a 100 mg/mL CsI solution.^36^

### Gel Electrophoresis

Proteins were analyzed by gel electrophoresis using 4–12% Bis-Tris gels (NuPAGE system, Thermo Scientific) at a constant voltage of 200 V for 35 min. The SeeBlue Plus 2 prestained protein marker (Thermo Scientific) was used. The gel was stained with InstantBlue Protein Stain (Expedeon).

### ATR-IR Spectroscopy

All IR measurements were performed on a Bio-ATR unit in a Vertex 70 IR spectrometer (BRUKER, Billercia, USA) equipped with a K10 thermostat (Thermo Fisher Scientific, Schwerte, Germany). Twenty microliters of the sample were placed on the zinc selenide crystal of the ATR-IR device, and the sample cell was sealed. A BIO-ATR experiment was performed with the software OPUS and the software package Protein Dynamics at a constant temperature of 37 °C and 256 accumulations. The spectra are difference spectra; the background spectra (of the buffered solution without the molecules of interest) were measured directly before and subsequently subtracted directly from the sample spectrum. The evaluation took place in Opus and Origin.^37^

### Small Angle X-ray Scattering

All SAXS measurements were performed in transmission mode in a SAXLAB laboratory configuration (Retro-F) with an AXO Microfocus X-ray source. The AXO Multilayer X-ray Optic (AXO Dresden GmbH, Dresden Germany) was used as a monochromator for Cu–Kα radiation (λ = 0.154 nm). The PILATUS3 R 300 K (DECTRIS, Baden, Switzerland) was used as a two-dimensional detector. 20 microliters of the sample were pipetted into a mark tube with a diameter of 1 mm and 0.01 thickness from Hilgenberg. The capillary was sealed and inserted into a temperature-controlled sample stage. The samples containing IL showed a high contrast and were measured for 3–5 h. The reference measurements of buffer and glass ran for 10 h and were used for background correction. The investigated temperatures ranged from 20 to 70 °C in 10 °C steps.^38^ Scattering intensities were corrected for background, transmission, and sample geometry and subsequently angular averaged and plotted versus the scattering angle *q* (*q* ranging from 0.02 to 0.7 A^–1^). All models used in fitting the data are explained in more detail in the Supprting Information, section 4.

### Protein Expression and Purification

A pet22b+ vector was used for the transformation of BL 21 cells. The preculture with the transformed cells was incubated overnight. For the main culture, 15 mL of the preculture was put into 1 L of LB media and the induction of the expression was started with IPTG after 3 h. Subsequently, the cells were lysed, and the protein was purified first with nickel affinity chromatography and second through ion exchange chromatography with a kta pure HPLC system. Thereafter, the protein was dialyzed against the refolding buffer and finally against deionized water. The last step was lyophilization of MBP from the solution.

## Results and Discussion

### Mixing-Ratio-Dependent Formation of Nanostructures

**Figure 1:**
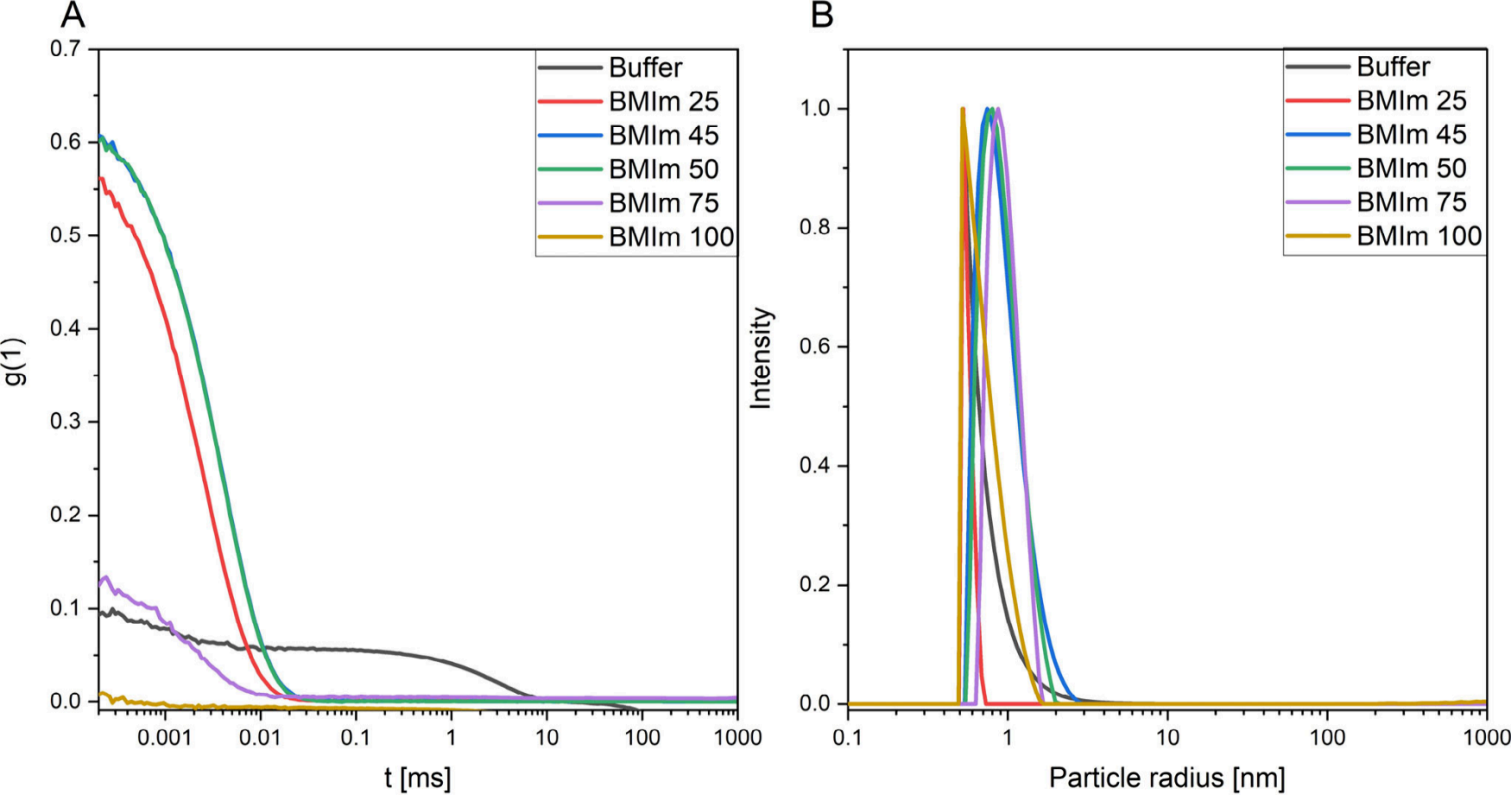
(A) DLS-based correlation function of HEPES buffer, BMIm 25, BMIm 45, BMIm 50, BMIm 75, and BMIm 100, where the number denotes the vol % of BMImBF_4_ within the aqueous mixture. (B) Corresponding particle size distributions derived from the correlation functions of Figure 1A. Note that in Figure 1A, the blue (BMIm 45) and green (BMIm 50) correlation functions are almost undistinguishable.

From our preliminary work, e.g., in Cerajewski et al.^39^ or Kattnig et al.,^21^ we know that EPR spectroscopy can be used to study intrinsic nanostructures in aqueous IL and deep eutectic solvent (DES) mixtures. As already reported in the literature, broadly scanning BMImBF_4_ miscibility in water, we found that BMIm is miscible with water at any ratio.^40,41^ It forms micellar or lamellar nanostructures in aqueous solution.^21^ We assume that these structures originate from local concentration differences triggered by the amphiphilic character and aqueous self-assembly of the BMIm cations and their ionic interactions with the anions. We found that these structures are ratio-dependent. In the preliminary tests, the most promising ratio to obtain rather homogeneous and small structures was 50:50 (vol:vol). To obtain a more precise value for the ratio or ratios that foster such nanostructures, dynamic light scattering (DLS) experiments of aqueous/ionic liquid (IL) mixtures with different BMIm ratios were performed, starting from 0 vol % up to 100 vol % BMImBF_4_ in 5 vol % steps. Figure 1A shows the autocorrelation functions of mixtures at selected mixing ratios. Figure 1B shows the corresponding particle size distributions.

The field autocorrelation function *g*(1) describes the time correlation of a scattering signal, and its characteristic decay time is proportional to the diffusion coefficient of the particle.^42−44^ The numerical amplitude value should be between 0 and 1, and from our experience we defined the significant threshold for structures to be considered to be 0.3. We refrain from the analysis of functional values below this significance threshold. When inspecting these initial DLS-derived correlation functions, the samples with the clearest scattering that deviates from homogeneous solution are the ones containing 45 and 50 vol % BMImBF_4_. We are well aware of the fact that standard analysis of light scattering techniques is model-based and that the particle size is calculated as an ideal sphere that moves with the same velocity as the measured particle.^23^ Hence, to further investigate that the nanostructured entities found in DLS are not measurement artifacts, we conducted small-angle X-ray scattering (SAXS) experiments with the most prominent mixing ratios of 0, 25, 45, 50, and 75 vol % BMIm.

Analogously to DLS, we analyzed the scattering profiles of the solutions by a model of uniform spherical scatterers. The model relies on the so-called Schultz sphere distribution, which is the most studied model distribution for polydisperse systems providing an average size in terms of the radius, *r*, and a polydispersity PD, defined as the relative distribution width of the radius. For the measurements, we obtained the following apparent radii: buffered solution, *r* = 0 ± 0.02 nm; BMImBF_4_ 25%, *r* = 1.40 ± 0.02 nm, BMImBF_4_ 45%, *r* = 1.52 ± 0.02 nm; BMImBF_4_ 50%, *r* = 1.67 ± 0.02 nm; and BMImBF_4_ 75%, *r* = 0.24 ± 0.02 nm with a PD = 0.2. It is visible that the measurements of 25, 45, and 50 vol % show nanostructure formation on the length scale of 1.5 nm for a wide range of concentrations, which is comparable to the DLS results. However, at BMIm concentrations of well above 50%, as in the case of BMIm 75 vol %, the structures are significantly reduced in size to ∼0.24 nm. As we aimed at exploring new applications for aqueous/IL mixtures based on their intrinsic nanostructure, we decided to use the aqueous 45 and 50 vol % BMImBF_4_ mixing ratios for tests as potential model-membrane systems. Hence, we added MBP, which has been amply characterized in-detail by our group in recent years, both in aqueous solution as well as in phospholipid-based structures like large unilamellar vesicles (LUVs), lipid nanodiscs, and phospholipid monolayers.^45,46^

### MBP and Aqueous IL Nanostructures

**Figure 2:**
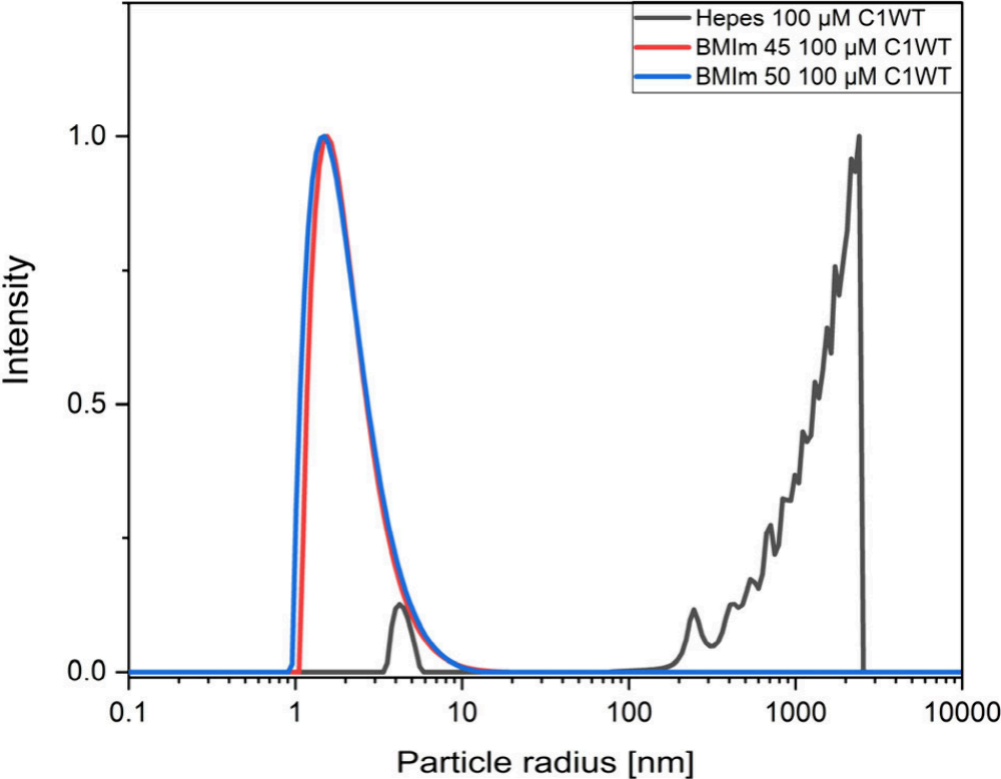
Apparent particle size distributions of BMIm 45% (red) and BMIm 50% (blue) with 100 μM MBP C1WT in comparison to 100 μM MBP C1WT dissolved in HEPES buffer.

After identifying water/IL mixing ratios promising to harbor well-defined nanostructures, further experiments were performed to test the behavior of MBP in these mixtures. To characterize the influence of aqueous/IL mixtures on MBP, first the DLS measurements, as a simple but meaningful technique to detect nanostructures, were repeated in the presence of MBP. Figure 2 shows the results of the particle size distributions of a sample that contains 45 and 50 vol % BMImBF_4_ and 100 μM rmMBP C1 WT. The respective size distributions of the double cysteine variant C1S17CH85C are almost identical to those of the WT and are shown in Figure S1.

It is apparent that a monodisperse, skewed particle size distribution with an average radius of 1.5 nm ranging from ∼1 to 10 nm emerges with MBP present, whereas the measurements on the aqueous protein samples without BMImBF_4_ reveal aggregates larger than 100 nm, known to originate from protein clusters and aggregates.^47^ In contrast, the rather narrow distribution up to 10 nm in the case of MBP in the BMIm solutions could reflect the distribution of individual macromolecules or low-molecular-weight oligomers in the aqueous/IL mixture instead. It seems straightforward to conclude that the nanostructures provided by the aqueous/IL mixtures may stabilize MBP in solution, potentially so that it can assemble at the interface between water-rich and IL-rich domains. To obtain further insights into the local microstructure of the system including MBP, we conducted EPR experiments using two different kinds of spin probes, TEMPO and 16-DSA.^48,49^ The reasoning to test the system with amphiphilic spin probes that self-assemble in the system through supramolecular/noncovalent interactions is to obtain insights into local nonpolar regions that might be formed by the BMIm cation and the protein.

The measured data, the simulation results, and the documentation of the evaluation parameters for both spin probes can be found in the SI. The results of the spectroscopic measurements with TEMPO in variable samples are as follows: for the HEPES-buffered samples, *a*_iso_ = 48.33 MHz and τ_corr_ = 16.66 ps, and for the BMIm 45 and 50, *a*_iso_ = 46.66 MHz and τ_corr_ = 16.66 ps. The hyperfine splitting constant *a*_iso_, which allows us to obtain information on the polarity of the spin probe surroundings, and the rotational correlation time τ can be used as specific parameters for the evaluation of the nanostructure around the spin probe. The larger the value of *a*_iso_, the greater the polarity in the surroundings.^29,50−52^ The decrease of *a*_iso_ can be explained with the nonpolar character of the butyl side chain of BMIm, while τ remains constant. The small and amphiphilic TEMPO molecule may stay at the internal interfaces between the organic and the water-rich regions of the mixtures. Since addition of MBP to the samples does not change the EPR spectra at all, one may safely assume that the spin probe does not directly interact with the protein. We then repeated the experiments using 16-DSA as a spin probe. 16-DSA is a modified fatty acid (stearic acid) and therefore much less water-soluble than TEMPO. For the HEPES-only based samples, we find *a*_iso_ = 44 MHz and τ_corr_ = 77 ps, and for the samples in 45% and 50% BMImBF_4_, *a*_iso_ = 42.75 MHz and τ_corr_ = 477 ps. The samples that contain BMImBF_4_ have understandably lower *a*_iso_ (lower polarity), as already seen for TEMPO, but also 6 times slower τ values. This indicates an increase in the viscosity due to the IL that is sensed by the stearic acid spin probe. As already seen in the TEMPO-probed samples, no differences with and without protein are observed for 16-DSA. Hence, 16-DSA seems to interact directly with the organic nanostructures in the aqueous IL mixtures, apparently being “built into” them. Overall, we can state that these initial measurements of aqueous/IL mixtures are consistent with the picture of formation of two domains, one water-rich and one IL-rich.^21,49^ We found no interactions between the respective spin probes and the protein, indicating that they may not directly encounter each other within the nanostructured regions. Therefore, we changed our approach from spin probing to spin labeling of MBP (i.e., to covalently link the nitroxide to the protein) to test the effect of the aqueous IL mixture on a membrane IDP like MBP.

### Spin Labeling of Recombinantly Expressed MBP

To label the protein with a nitroxide radical directly, recombinantly produced MBP variants are needed. Commercially available bMBP is a mixture of different isoforms, and rmC1WT cannot be spin-labeled selectively. Accordingly, the 18.5 kDa C1 isoform with a net charge of +19 is also found to be the most abundant isoform in commercially available bMBP, while other isoforms were lower in abundance (Figure S2). So, for our next steps, besides the high charge isoform rmC1WT, a double cysteine variant C1S17CH85C was produced. Both are recombinant murine, rm, MBP variants, which are seen as being also typical for the human form. These proteins were chosen because the C1 form represents the “healthy” 18.5 kDa MBP isoform. The cysteine-containing variants at positions S17C and H85C have in the past been used for structural studies after double spin-labeling at these positions.^15,53^ In particular, position 85 is in an α-helical region at least when folded in LUVs. The proteins were expressed and purified as described in the Materials and Methods section. The protein identification was carried out using mass spectrometry (see the Materials and Methods). The sequence analysis via MaxQuant resulted in a sequence coverage of 88% for C1WT and 75% for C1S17CH85C. To further confirm the identity, MascotSearch was used and gave a sequence coverage of 75% for C1WT and 63% for C1S17CH85C.^35^

To check the purity, we performed ATR-IR measurements.^54^ The results of these measurements can be found in the SI. We used bMBP as a standard to compare its IR bands with the bands of the recombinantly produced variants. We find the typical protein bands: (i) the amide A band at 3070 to 3300 cm^–1^, which is derived from the N–H stretching vibrations;^26,55^ (ii) the typical amide I band from 1600 to 1700 cm^–1^ from stretching vibrations of C–O bonds;^26,55^ and (iii) the amide II band from 1510 to 1580 cm^–1^, which stems mainly from in-plane N–H bending and CN stretching vibrations.^26,55^ The shape of the bands is identical for each sample; only their intensities vary. Given the fact that bMBP is a mixture of variants (MS data in the SI), from this finding we conclude that all the proteins are indeed variants of MBP.

### Development of a Model Membrane System

We have now set the reference framework for assessing the IL/water mixtures and will in the following focus on how the mixtures affect the protein structure of MBP, first through CW EPR spectroscopy of spin-labeled C1S17CH85C.

**Figure 3:**
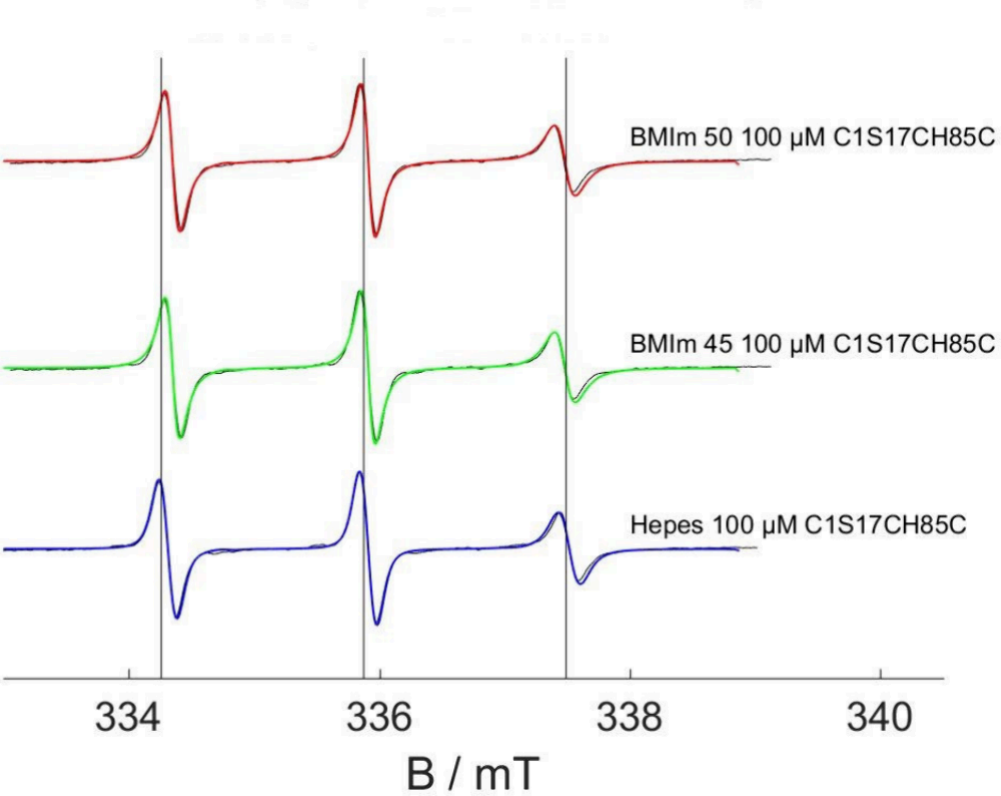
EPR spectra (black) and simulations (color) of doubly spin-labeled C1S17CH85C in aqueous, HEPES-buffered mixtures with BMImBF_4_ at 0% (blue, bottom), 45% (green, center), and 50% (red, top), respectively.

Figure 3 shows the CW EPR spectra and the results of the simulations, which gave *a*_iso_ = 45 MHz and τ_corr_ = 366 ps for the HEPES-based samples and *a*_iso_ = 44.33 MHz and τ_corr_ = 209 ps for the 45% and 50% aqueous BMImBF4-based samples. The spectral shape and simulations are congruent with spin labels whose rotation around the *z*-axis in the *g*-tensor frame is restricted. This is a result of the geometry of the spin label’s chemical attachment to the protein.^56^ The values of τ of the samples prepared with BMImBF_4_ reveal faster rotational motion than in the samples without IL. This could indicate that the protein is truly dissolved and fully solvated in the aqueous/IL mixtures and is not aggregated or clustered like in purely aqueous solutions. Furthermore, from comparison of the *a*_iso_ values, one can deduce that the chemical environments of the spin labels in IL/water-based samples (*a*_iso_ = 44.33 MHz) are less polar than in pure buffered solution (*a*_iso_ = 45 MHz). One may interpret the findings for *a*_iso_ and τ_corr_ together as a sign that the protein accumulates individually and is solvated (faster τ) either inside the IL rich region or at the interface between the IL-rich regions (lower *a*_iso_) and the surrounding water. To test this hypothesis, we used four-pulse double electron–electron resonance (DEER), a pulse EPR technique that allows determining dipolar interactions between electron spins and obtaining distance distributions. Such insights into the nanoscopic structure of the structured liquid may be available through understanding of the protein’s conformations on the molecular level.^57−59^

**Figure 4:**
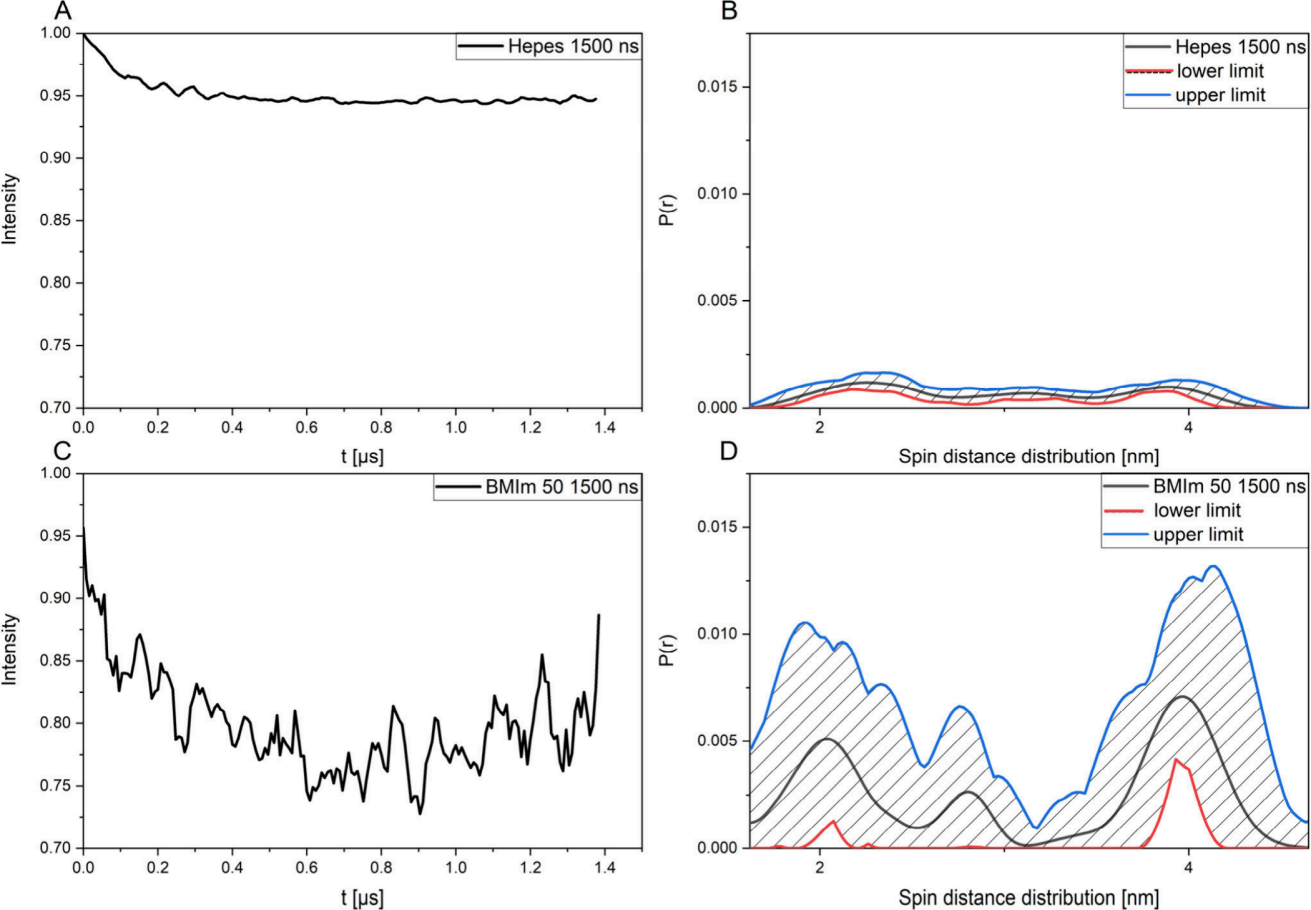
(A) Background-corrected time traces of rmC1S17CH85 in HEPES-buffered solution with 1500 ns measurement time. (B) The HEPES-associated particle size distribution validated with DEER analysis. (C) Background-corrected time traces of rmC1S17CH85 in aqueous BMImBF_4_ 50% with 1500 ns measurement time. Note that the apparent difference in the noise scale of the time traces in A and C stems from increased so-called “proton modulation” of the unpaired electron spin to methyl ^1^H nuclear spins (from the organic BMIm ion) in the case of rmC1S17CH85 in the aqueous BMImBF_4_ 50% solution. (D) The BMImBF_4_ 50% associated particle size distribution validated with DEER analysis. Note the large difference in modulation depths between the time traces in A and C and the distribution weights in B and D, respectively.

Figure 4A shows the measured time trace of 100 μM of the doubly spin-labeled rmC1 S17CH85C in HEPES buffer, and Figure 4B shows the corresponding validated spin distance distribution. Figure 4C shows the measured time trace of 100 μM of the doubly spin-labeled rmC1 S17CH85C in the 50% aqueous IL mixture. Figure 4D shows the corresponding validated spin distance distribution. Remarkably and unlike the reference measurements in pure HEPES buffered aqueous solution (Figures 4A and 4B), in the aqueous IL mixture we find large modulation depths in the time traces, 0.23 vs 0.05 in pure buffered solution (as observable in Figures 4A and 4C). This is reflected in high weights in the distance distributions that indicate a pattern in the spin distance distributions resembling a trimodal distribution (∼1.8, ∼2.8, and ∼3.8 nm) found for singly labeled MBP C1 (and rmC8) in myelin-like LUVs.^31,53^ Unlike the distributions of the singly spin-labeled variants, though, the peak at short distances (∼1.8 nm) is more strongly populated, which may indicate that the *intra*molecular spin distance distribution of the doubly spin-labeled rmC1S17CH85C variant contributes at the short distances to the overall *inter*molecular dominated distributions.

Taken together, the published results and our measured data strongly hint toward a self-assembly process of MBP molecules taking place on the molecular level. Moreover, this self-assembly in the IL-containing aqueous mixture cannot be considered a trivial aggregation or something like solvation in a denatured conformation. Hence, one may conclude that the nano/microstructure provided by aqueous/BMImBF_4_ mixtures induce MBP to fold and self-assemble similarly to myelin-like phospholipid vesicles, which are generally accepted as excellent models for the cytoplasmic leaflet of the myelin sheet. Hence, the nanostructure is also different, more “native”, in the IL/water mixtures than in aqueous surfactant micelles, which of course are often used as simple models of lipid bilayers. To further probe the system with the protein on length scales larger than the DEER/EPR scale of up to 5 nm, we conducted temperature-dependent SAXS measurements of samples that contained 50 vol % BMImBF_4_ with and without 285 μmol MBP.^21^

Please note that all details on the used fitting models are explained in detail in the Supporting Information.

**Figure 5:**
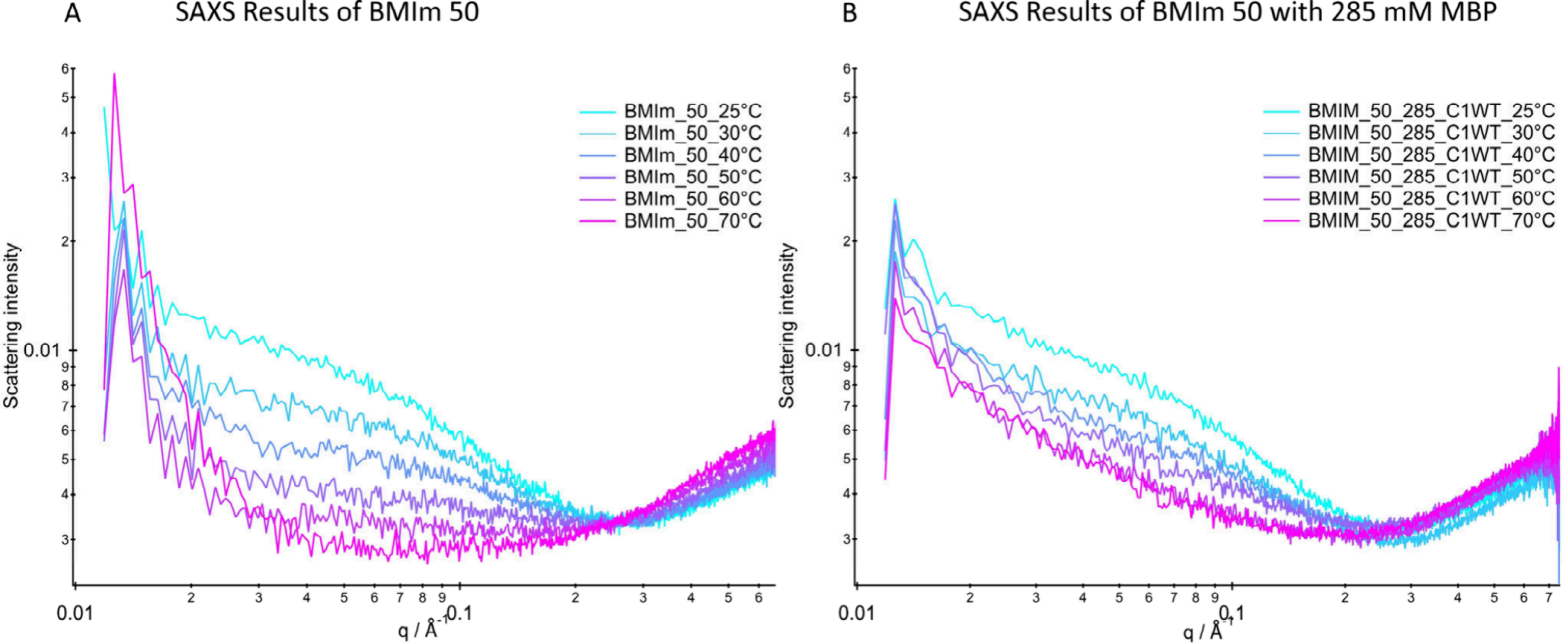
Temperature-dependent scattering profiles of BMImBF_4_ 50% without (A) and with (B) 285 μM of MBP C1WT. Note that the values below 0.015 A^–1^ were not recorded due to a beam stopper.

Figure 5A first shows the results of the BMIm solution without protein for the temperature range 25–60 °C. It is apparent that the intensity of the scattering at low *q* values is reduced with increasing temperatures. This reduction may hint toward better mixing of the components, becoming more like samples with a majority component of BMIm, e.g., the 75% sample (Figure S4), in which the minority component (water) is dissolved. Indeed, also the apparent sizes of the structures found in the analytical sphere model discussed above shrink to the point of vanishing. The vanishing structures can be explained with the increased miscibility of the molecules. As described above, the nanostructures arise from local concentration differences derived from the difference in polarity between BMImBF_4_ and water. Because of the higher temperature, this nanoscopic segregation becomes increasingly dynamic and both phases mix, resulting in a homogeneous solution.^60,61^

In contrast, upon addition of MBP, the scattering curve of the mixture with MBP in Figure 5B displays a less pronounced temperature effect. The obtained particle size shows an increase in size with higher temperatures from ∼4.9 nm at 25 °C to values >100 nm at 60 °C. However, the model that best reproduces the scattering curves is now a string of individual, threaded beads (for more detail see section 4 of the SI). Hence it can be concluded that with increasing temperature, the process leads to a growth of structures similar to beads on a string.

To derive a coherent picture of the self-assembly processes in the complex mixture of BMImBF_4_, water, and MBP, it is necessary to combine all findings so far. From the DLS and more accurately from SAXS measurements, an average size of the liquid phases and their connectivity was obtained. CW-EPR experiments with the spin probes TEMPO and 16-DSA indicate that nanostructures are provided by aqueous/IL mixtures that originate from local concentration differences, which arise due to the amphiphilic character of the IL.

It is safe to interpret the findings such that through the interaction of MBP with the 45% or 50% aqueous BMImBF_4_ mixtures, the protein is dissolved without clustering or aggregation. This in itself is noteworthy, although one may imagine that this apparently solvated state of MBP may simply not be the native state or a related state in solution, as the membrane IDP MBP needs a myelin-like phospholipid membrane to attain the native state. It is therefore decisive to obtain insights into the structure or self-assembled state of MBP in the mixture. To this end, double electron–electron resonance (DEER), a pulse EPR method to determine intra- and overlaid intermolecular distance distributions between electron spins (on spin labels), was employed and indicates a self-assembly process similar to that in myelin-like LUVs.

Therefore, MBP may assemble at an interface between hydrophilic/charged and nonpolar environments. This interface likely, when realistically viewing the system, stems from the nanostructures formed between the IL-rich and surrounding water-rich domains. The contrast between the hydrated and charged “surface layer” and nonpolar alkyl layer remarkably seems to be strong enough to allow rmC1MBP to self-assemble like in a phospholipid bilayer membrane.

**Figure 6:**
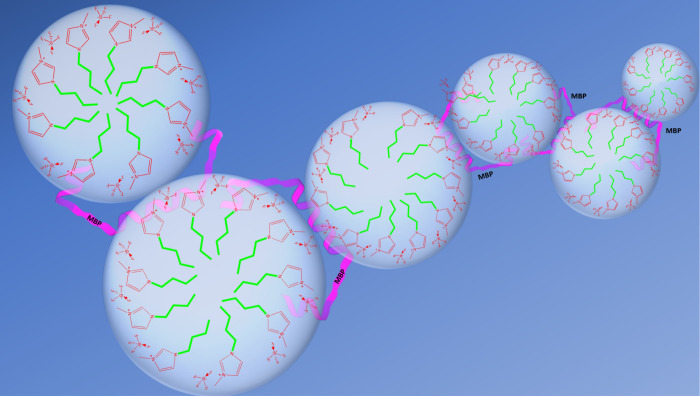
Schematic representation of the membrane model structure of ionic liquid (green and red in light blue micelle) in water (dark blue) and their suspected interaction with MBP (magenta) with the provided structure. MBP is assumed to be in the “paper clip” structure that was deduced from several studies before to be found in myelin-like LUVs.

Finally, SAXS measurements indicate that on a still nanoscopic level but larger than on the level of individual protein molecules, MBP induces an ordering effect of the phase-separated IL compartments similar to beads on a string. Hence, the aqueous BMImBF_4_ mixtures at the peculiar values of 45% and 50% provide a nanomicellar structure with a hydrodynamic radius ranging from 1.5 to 5 nm. Unlike ordinary surfactant micelles, these structures have a stabilizing effect on MBP and apparently do not only dissolve it but even trigger a self-assembly process in the protein, allowing it to assemble like in the myelin sheath. MBP is known to have three secondary structure units that have a high propensity to fold into short α-helical regions in a phospholipid membrane. Furthermore, there is ample evidence in prior studies that the three α-helical regions fold into a “paper-clip-like” structure in myelin-like LUVs.^14−18^ Although we cannot finally prove the formation of the α-helices, the DEER data indicate self-assembly similar to that in LUVs. Since the self-assembly process usually requires individually properly folded proteins, one may infer that the MBP molecules are folded “correctly”. We have indicated this in the schematic representation in Figure 6, where paper-clip-type MBPs interact with the IL-based micelles in the background of water. More experiments to obtain clear evidence in particular for the α-helical regions need to be conducted in future work.

Furthermore, SAXS data strongly indicate a stacking process similar to a threading of a pearl necklace, which may be interpreted as also shown schematically in Figure 6: individual IL-rich nanostructures are threaded together at higher temperature by MBP molecules that self-assemble as they do in a phospholipid bilayer membrane. As such, they shape the nanosegregated regions into a simple pseudomembrane model system.

## Supporting Information Available

Additional EPR measurements, SAXS measurements, IR measurements, and DLS measurements (PDF)

## Abbreviations

16-DSA: 16-doxyl stearic acid
ATR-IR: attenuated total reflection infrared spectroscopy
BMIm: 1-butyl-3-methylimidazoliumtetrafluoroborate
C1S17CH85C: C1 wildtype substituted at positions 17 and 85 with cysteine
C1WT: C1 wildtype
cw-EPR: continuous wave electron paramagnetic resonance
DEER: double electron–electron resonance
DLS: dynamic light scattering
IDP: intrinsically disordered protein
IDR: intrinsically disordered region
MBP: myelin basic protein
MS: mass spectrometry
MTSSL: (2,2,5,5-tetramethyl-3-((2methyl-2,2-dioxo-2λ^6^-disulfan-1-yl)methyl)-2,5-dihydro-1H-pyrrol-1-yl)oxyle
MAXS: middle angle X-ray scattering
SAXS: small angle X-ray scattering
TEMPO: 2,2,6,6-tetramethylpiperidinyloxyl
WAXS: wide angle X-ray scattering
