# Supplementary material for: Aqueous Ionic Liquid Mixtures as Minimal Models of Lipid Bilayer Membranes
Source: ACS Biomater Sci Eng. 2024 Jul 27;10(8):4802–11. doi: 10.1021/acsbiomaterials.4c00740 (PMC11322907; doi:10.1021/acsbiomaterials.4c00740)
Supplement: Supplementary file 1 — ab4c00740_si_001.pdf [file ab4c00740_si_001.pdf]

Aqueous Ionic Liquid Mixtures as Minimal Model of Lipid Bilayer Membranes

Jonas Volmer[1], Ulrike Cerajewski[1], Marie Alfes[2,#], Julian Bender[2,$], Josefin Abert[1], Carla Schmidt[2,3], Maria Ott[4], and Dariush Hinderberger[1,2]*.

1. Martin Luther University Halle-Wittenberg, Institute of Chemistry, Physical Chemistry – Complex Self-Organizing Systems, Von-Danckelmann-Platz 4 06120 Halle (Saale), Germany

2. Interdisciplinary Research Centre HALOmem, Institute of Biochemistry and Biotechnology, Charles Tanford Protein Centre, Martin Luther University Halle-Wittenberg, Kurt-Mothes-Str. 3a, 06120 Halle, Germany;

3. Department of Chemistry ─ Biochemistry, Johannes Gutenberg University Mainz, Biocenter II, Hanns-Dieter-Hüsch-Weg 17, 55128 Mainz, Germany

4. Martin Luther University Halle-Wittenberg, Institute of Biochemistry and Biotechnology, Protein biochemistry, Kurt-Mothes-Str. 3 06120 Halle (Saale), Germany

# - Supporting Information-

1. Additional DLS Measurements

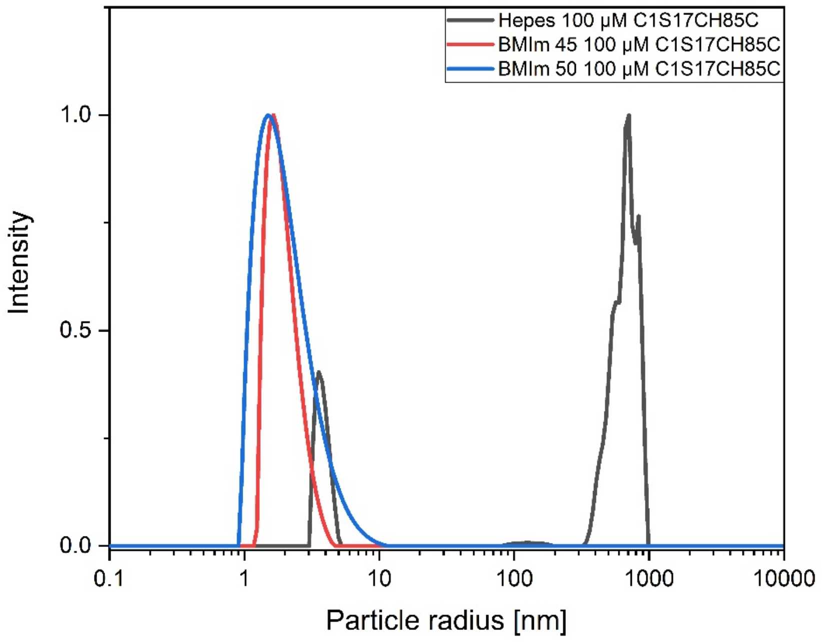

Figure S1 DLS Measurements of MBP rmC1S17CH85C.

2. Results of Mass spectrometry

Table S1 Identification of MBP_C1WT by LC-MS/MS. Max Quant search results are given for the protein and for peptide sequences.

Protein information					
**Protein**	**MW [Da]**	**Sequence coverage [%]**	**Protein score**	**MS/MS count total**	**# Peptide sequences**
MBP_C1WT	19,236	88	323.31	1348	41

Peptide information				
**Peptide sequences**	**Start position**	**End position**	**MQ score**	**MS/MS count**
DSHTRTTHYGSLPQK	57	71	145.83	3
DTGILDSIGR	32	41	252.72	48
DTGILDSIGRFFSGDR	32	47	164.15	8
DTGILDSIGRFFSGDRGAPK	32	51	335.06	10
FFSGDRGAPK	42	51	168.74	155
FFSGDRGAPKR	42	52	148.5	2
FSWGAEGQK	111	119	143.12	5
FSWGAEGQKPGFGGYGGR	111	128	199.8	31
GAYDAQGTLSK	141	151	149.72	61
GAYDAQGTLSKIFK	141	154	180.2	24
GAYDAQGTLSKIFKLGGR	141	158	147.33	13
GFKGAYDAQGTLSK	138	151	318.47	90
GFKGAYDAQGTLSKIFK	138	154	126.95	9
GLSLSRFSWGAEGQK	105	119	88.187	4
GRGLSLSR	103	110	166.82	3
HGFLPRHRDTGILDSIGR	24	41	188.27	12
HRDTGILDSIGR	30	41	315.72	154
HRDTGILDSIGRFFSGDR	30	47	284.48	12
IFKLGGR	152	158	142.63	12
IFKLGGRDSR	152	161	219.19	7
LGGRDSR	155	161	57.335	1
NIVTPRTPPPSQGK	89	102	198.97	3
NIVTPRTPPPSQGKGR	89	104	105.66	3
PGFGGYGGR	120	128	118.69	3
RGSGKDSHTR	52	61	37.18	1
SAHKGFKGAYDAQGTLSK	134	151	359.6	16
SGSPMAR	162	168	114.59	2
SKYLATASTMDHAR	10	23	355.26	120
SKYLATASTMDHARHGFLPR	10	29	148.15	14
SQHGRTQDENPVVHFFK	72	88	188.96	22
SQHGRTQDENPVVHFFKNIVTPR	72	94	130.65	26
TPPPSQGK	95	102	89.142	11

TPPPSQGKGRGLSLSR	95	110	165.33	11
TQDENPVVHFFK	77	88	242.97	104
TQDENPVVHFFKNIVTPR	77	94	358.52	42
TQDENPVVHFFKNIVTPRTPPPSQGK	77	102	259.82	10
TTHYGSLPQK	62	71	149.82	165
TTHYGSLPQKSQHGR	62	76	226.73	11
TTHYGSLPQKSQHGRTQDENPVVHFFK	62	88	142.22	17
YLATASTMDHAR	12	23	264.3	87
YLATASTMDHARHGFLPR	12	29	135.45	16

Table S2 Identification of MBP_C1S17CH85C by LC-MS/MS. Max Quant search results are given for the protein and for peptide sequences.

Protein information					
**Protein**	**MW [Da]**	**Sequence coverage [%]**	**Protein score**	**MS/MS count total**	**# Peptide sequences**
MBP_C1S17CH85C	19,252	74.9	323.31	215	22
**Peptide information**					
**Peptide sequences**		**Start position**	**End position**	**MQ score**	**MS/MS count**
DTGILDSIGR		32	41	199.02	31
DTGILDSIGRFFSGDR		32	47	89.466	2
DTGILDSIGRFFSGDRGAPK		32	51	161.86	2
FFSGDRGAPK		42	51	95.775	20
FSWGAEGQKPGFGGYGGR		111	128	92.834	2
GAYDAQGTLSK		141	151	134.25	7
GFKGAYDAQGTLSK		138	151	78.401	6
GRGLSLSR		103	110	157.68	3
HRDTGILDSIGR		30	41	160.67	37
HRDTGILDSIGRFFSGDR		30	47	24.594	2
IFKLGGR		152	158	46.351	0
IFKLGGRDSR		152	161	83.499	4
NIVTPRTPPPSQGK		89	102	83.844	1
PGFGGYGGR		120	128	49.358	1
SAHKGFKGAYDAQGTLSK		134	151	61.495	4
SKYLATACTMDHAR		10	23	107.99	8
TPPPSQGKGRGLSLS R		95	110	106.52	7
TQDENPVVHFFK		77	88	80.623	43
TQDENPVVHFFKNIVTPR		77	94	68.567	5
TTHYGSLPQK		62	71	150.36	14
TTHYGSLPQKSQHGR		62	76	150.26	2
YLATACTMDHAR		12	23	209.6	14

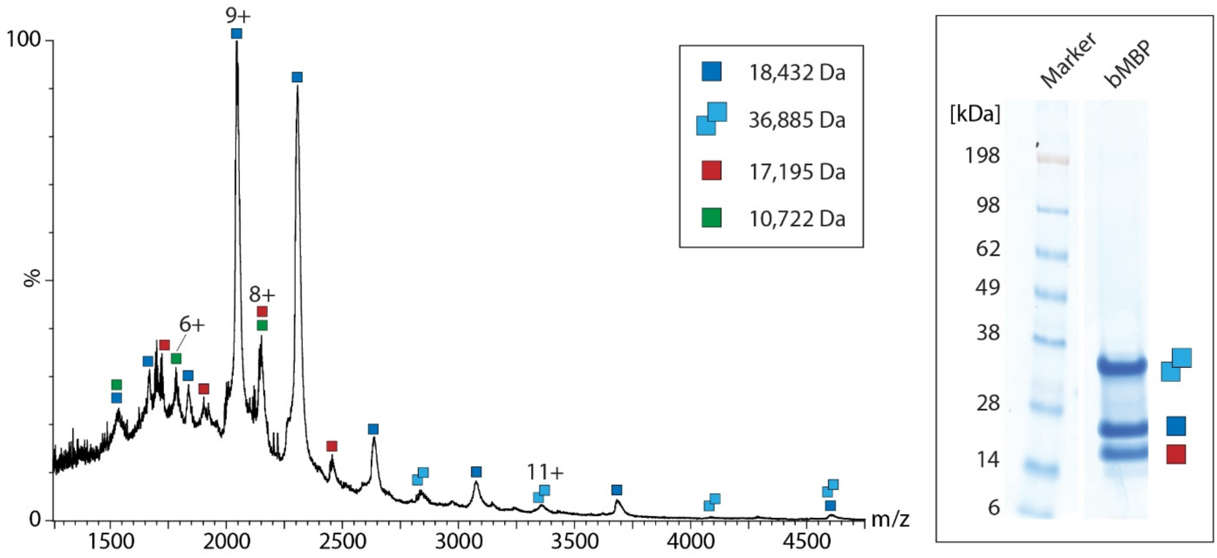

Figure S2 Native MS Spectrum and SDS Page of bMBP

The masses 18.4 kDa matches the C1 monomer + salt, 36.9 kDa matches the dimer and 17.2 kDa and 10.7 kDa matches the isoforms.

3. IR Results

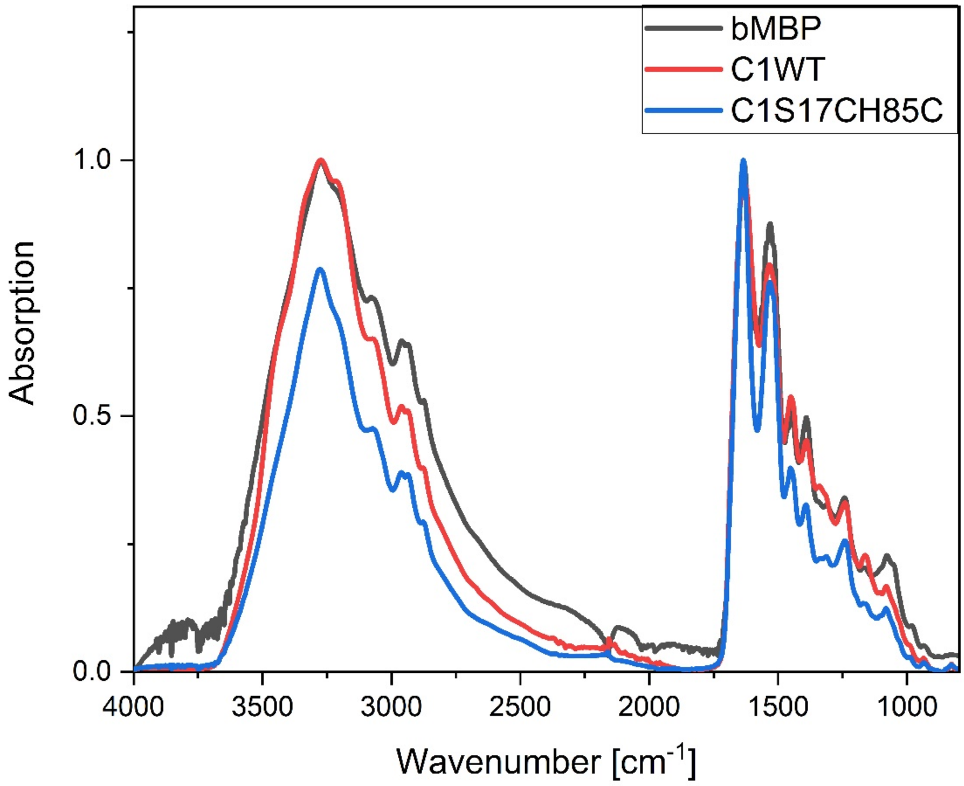

Figure S3 IR measurements of bMBP, rmMBP C1WT and C1S17CH85C

4. Documentation SAXS

Spheres with Schulz Size Distribution calculates the scattering for a polydisperse population of spheres with uniform scattering length density. The distribution of radii is a Schulz distribution. The Intensity is normalized by the average particle volume such that I(q) = scale*<F*F>/<Vol> + bkg, where F is the scattering amplitude of a sphere and the < > denote an average over the size distribution

Table S3 Input variables and default values of schulz sphere model

Parameters_sch	Coef_sch
Volume fraction	0.01
Mean radius	15
polydispersion	0.2
SLD sphere	$1*10^{-6}$
SLD solvent	$3*10^{-6}$
Background	0.001

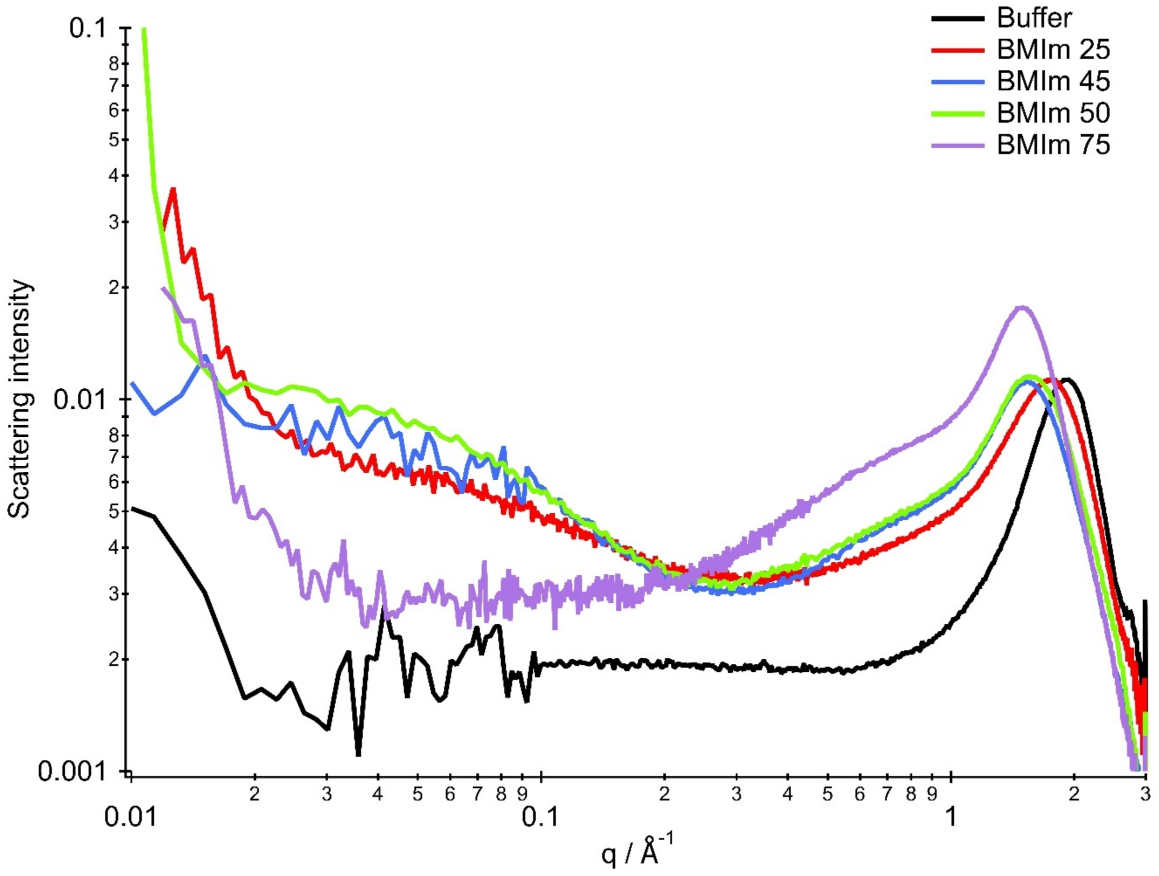

Figure S4 SAXS results for HEPES Buffer, BMIm 25, BMIm 45, BMIm 50 and BMIm 75

The data analysis was carried out with IgroPro7 as a mathematic model of fractal with polydisperse cylinders was chosen. Plots scattering from a mass fractal object using the model of Teixeria with a rigid cylinder (polydisperse radius) as the "building block". The radius in the fractal S(q) is the Rg of the cylinder. As long as the fractal length scale (correlation length) and the Rg of the chain are very different so that fractal knows no details of the cylinder structure, then this construct is valid. The returned value is the scattered intensity, on absolute scale of 1/cm. The model combines the scattering of the cylinder model and the fractal models. The "block radius" of the fractal model now

corresponds to the radius of gyration of the cylinder, which is: $R_g^2 = \frac{R^2}{2} + \frac{L^2}{12}$. The Input variables and the default values are in the following table:

Table S4 Input variables and default values fractal with polydisperse cylinders

Parameters_fraCyl	Coef_fraCyl
Volume fraction	0.05
Fractal dimension	2
Correlation length	500
SLD cylinder	$2e^{-6}$
SLD solvent	$6,35e^{-6}$
Cylinder radius	15
Cylinder length	200
Polydisp. of Radius	0.2
Background	0

Table S5 Results of the calculated Parameters for the SAXS-Temperature series of BMIm 50

T [°C]	Fractal dimension	Correlation Length [nm]	Particle Radius [nm]
25	1.01025	5.68	1.67
30	1.01025	4.98	1.67
40	1.01025	2.94	1.67
50	1.00266	1.91	1.67
60	1.00217	1.06	1.67
70	1.00217	0.69	1.67

Table S6 Results of the calculated Parameters for the SAXS-Temperature series of BMIm 50 with 285µM MBP

T[°C]	Fractal dimension	Correlation Length [nm]	Particle Radius [nm]
25	1.00009	4.96	1.67
30	1.00020	7.67	1.67
40	1.00251	7.87	1.67
50	1.06033	230.69	1.67
60	1.26824	$6.16*10^9$	1.67
70	1.26824	$6.17*10^9$	1.67

5. Documentation EPR

5.1. CW-EPR

The parameter set was used in the script both for aqueous solutions and for samples containing IL. In addition, the Euler angle had to be implied. The standard angle set for α, β and γ of [0 50 0] was used for the simulation. For the simulations, mainly the values of the hyperfine coupling tensor A and the rotational diffusion tensor D were adjusted. The isotropic value a_{iso} was calculated from the simulated tensor data using the following equation:

$$a_{iso} = \frac{(A_{xx} + A_{yy} + A_{zz})}{3}$$

Using a Brownian diffusion model based on the rotational diffusion tensor D, the rotational correlation time τ_{corr} was calculated according to the following equation:

$$\tau_{corr} = \frac{1}{6\sqrt[3]{D_{xx}D_{yy}D_{zz}}}$$

The parameters for the simulations can be found in the following tables.

Table S7 A-tensors and D-Tensors for samples containing TEMPO.

Sample	A_{xx}	A_{yy}	A_{zz}	D_{xx}	D_{yy}	D_{zz}
Hepes+TEMPO	25	15	105	$1*e^{11}$	$1*e^{10}$	$1*e^{9}$
Hepes+TEMPO+MBP	25	15	105	$1*e^{11}$	$1*e^{10}$	$1*e^{9}$
BMIm 45+TEMPO	25	15	100	$1*e^{11}$	$1*e^{10}$	$1*e^{9}$
BMIm 45+TEMPO+MBP	25	15	100	$1*e^{11}$	$1*e^{10}$	$1*e^{9}$
BMIm 50+TEMPO	25	15	100	$1*e^{11}$	$1*e^{10}$	$1*e^{9}$
BMIm 50+TEMPO+MBP	25	15	100	$1*e^{11}$	$1*e^{10}$	$1*e^{9}$

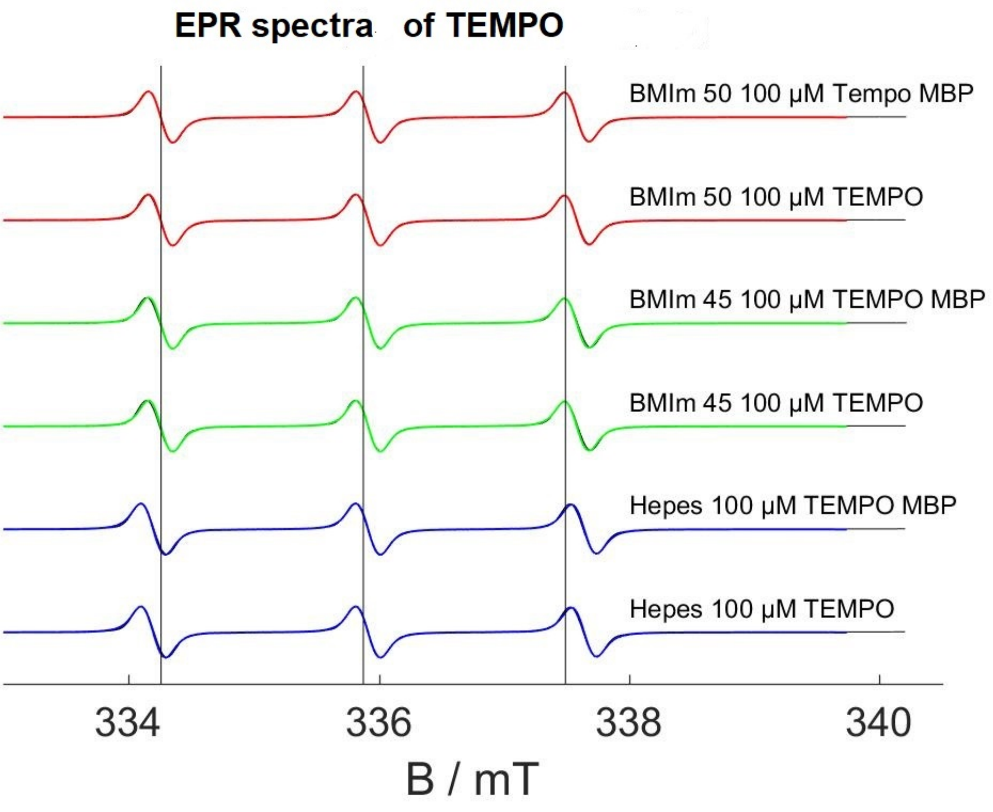

Figure S5 EPR-spectra (black) and simulations of HEPES, BMIm 45 and BMIm 50 with and without MBP using TEMPO as a spinprobe

Table S8 Results TEMPO

Sample	a_{iso}[MHz]	τ [ps]
HEPES	48,33	16,66
HEPES + MBP	48,33	16,66
BMIm 45	46,66	16,66
BMIm 45 + MBP	46,66	16,66
BMIm 50	46,66	16,66
BMIm 50 + MBP	46,66	16,66

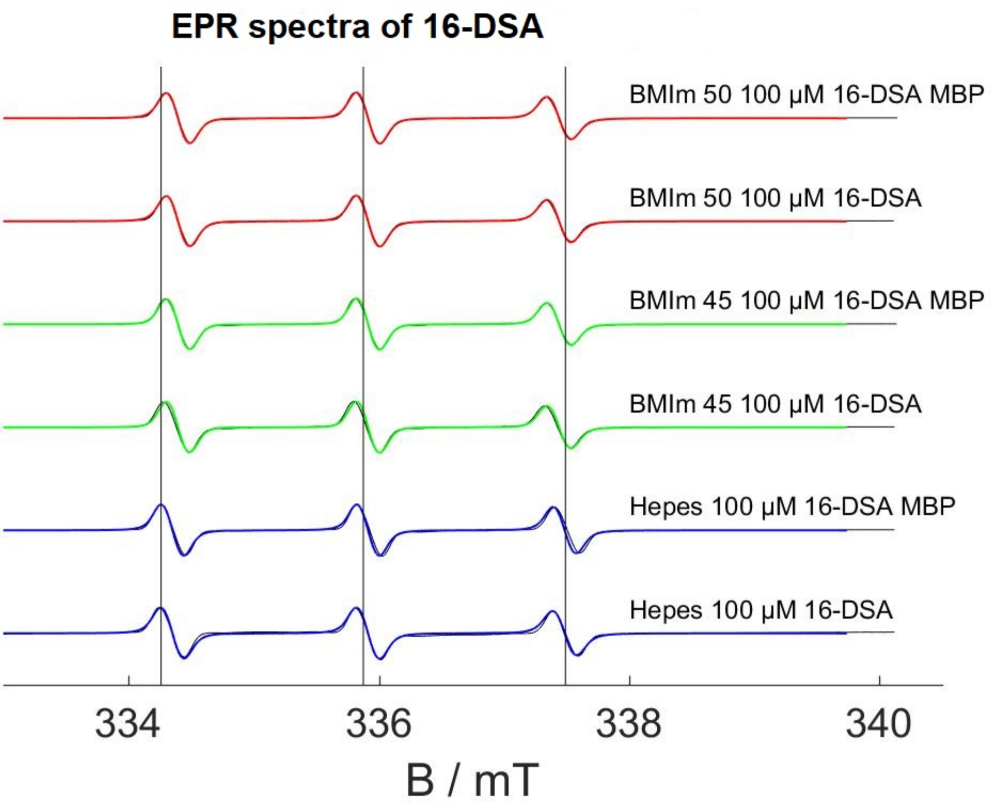

Figure S6 EPR-spectra (black) and simulations of HEPES, BMIm 45 and BMIm 50 with and without MBP using 16-DSA as a spinprobe

Table S9 A-tensors and D-Tensors for samples containing 16-DSA.

Sample	A_{xx}	A_{yy}	A_{zz}	D_{xx}	D_{yy}	D_{zz}
Hepes+16-DSA	17	15	100	$1*e^9$	$1*e^9$	$1*e^{10}$
Hepes+16-DSA+MBP	18	18	96	$1*e^9$	$5*e^9$	$6*e^9$
BMIm 45+16-DSA	17,25	15	96	$1*e^7$	$5*e^8$	$8.5*e^9$
BMIm 45+16-DSA+MBP	17,25	15	96	$1*e^7$	$5*e^8$	$8.5*e^9$
BMIm 50+16DSA	17,25	15	96	$1*e^7$	$5*e^8$	$8.5*e^9$
BMIm 50+16-DSA+MBP	17,25	15	96	$1*e^7$	$5*e^8$	$8.5*e^9$

Table S10 Resuls 16-DSA

Sample	a_{iso}[MHz]	τ [ps]
HEPES	44,00	77,00
HEPES + MBP	44,00	53,60
BMIm 45	42,75	477,00
BMIm 45 + MBP	42,75	477,00
BMIm 50	42,75	477,00
BMIm 50 + MBP	42,75	477,00

Table S11 A-tensors and D-Tensors for samples with spin labled C1S17CH85C

Sample	A_{xx}	A_{yy}	A_{zz}	D_{xx}	D_{yy}	D_{zz}
Hepes+C1S17CH85C	25	10	100	$1.5*e^8$	$5*e^8$	$1.25*e^9$

BMIm 45+ C1S17CH85C	20	15	97	$5*e^9$	$1*e^9$	$1*e^8$
BMIm 50+C1S17CH85C	20	15	97	$5*e^9$	$1*e^9$	$1*e^8$

Table S13 Results of the simulated Parameters for spinlabeled C1S17CH85C

Sample	a_{iso}[MHz]	τ [ps]
HEPES	45,00	366,00
BMIm 45	44,33	209,00
BMIm 50	44,33	209,00

5.2. Pulse EPR

Pulse EPR data were analyzed with the software package DeerAnalysis (Version 2019). The results were validated between the following parameters.

Table S14 Validation parameters of DEER Analysis

Parameters	Min.	Max.
White noise	0,02	1,50
Background start	240	1000
Background dim.	2,80	3,20
Background density	1,50	2,30
Modulation depth	0,175	0,260

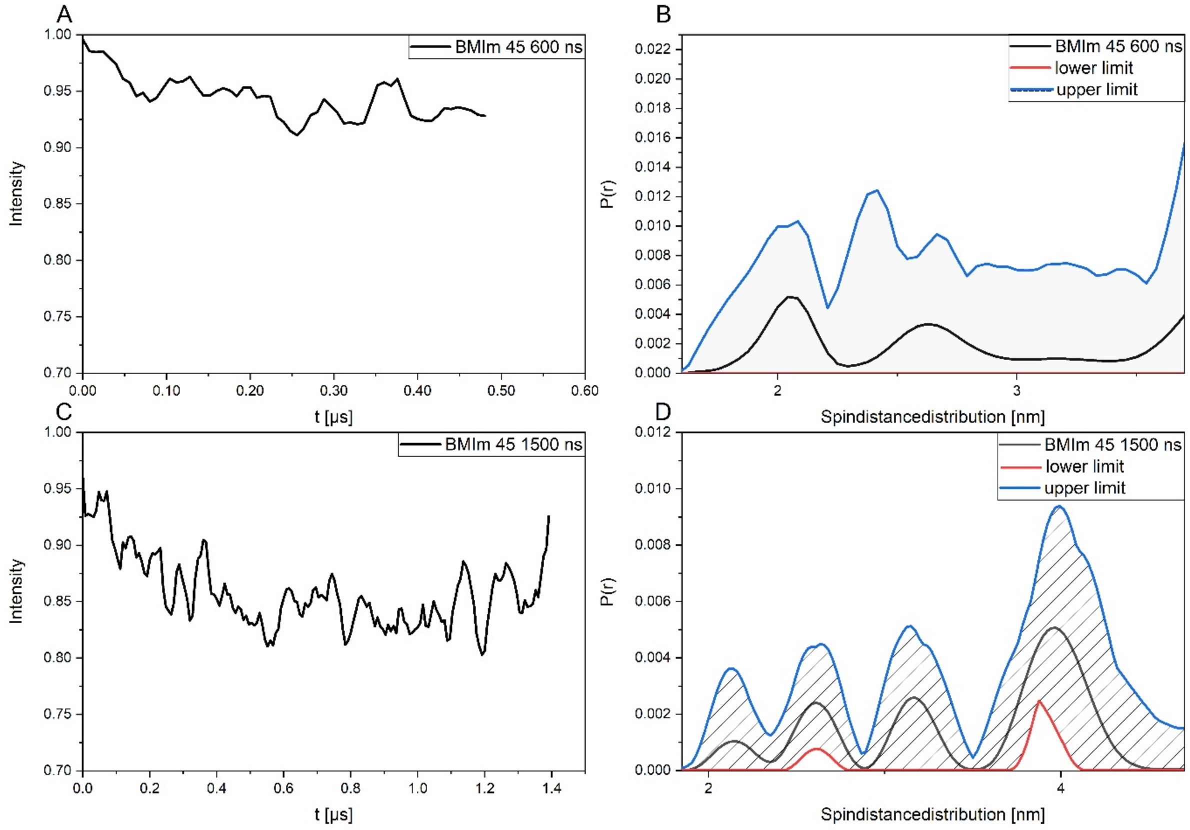

Figure S7 A: Time traces of rmC1S17CH85 in aqueous BMImBF$_4$ 45% solution with 600 ns measurement time; B: The BMImBF$_4$ 45% associated particle size distribution validated with DEER Analysis C: Time traces of rmC1S17CH85 in aqueous BMImBF$_4$ 45% with 1500 ns measurement time D: The BMImBF$_4$ 45% associated particle size distribution validated with DEER Analysis

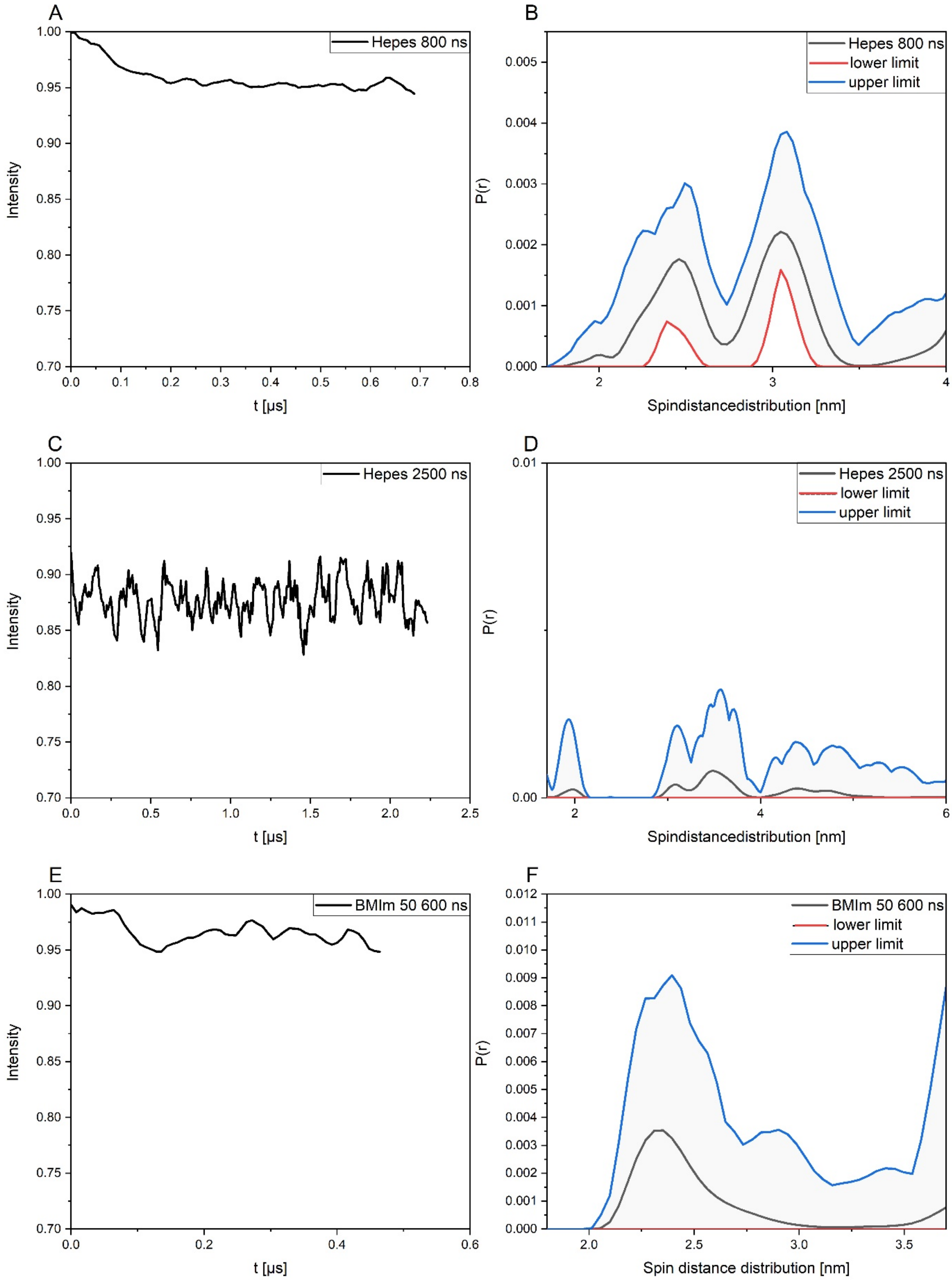

Figure S8 A: Time traces of rmC1S17CH85 in Hepes buffered solution with 800 ns measurement time; B: The Hepes associated particle size distribution validated with DEER Analysis C: Time traces of rmC1S17CH85 in Hepes buffered solution with 2500 ns measurement time; D: The Hepes associated particle size distribution validated with DEER Analysis E: Time traces of rmC1S17CH85 in aqueous BMImBF$_4$ 50% with 600 ns measurement time F: The BMImBF$_4$ 50% associated particle size distribution validated with DEER Analysis
